# Muscular Fitness Mediates the Association between 25-Hydroxyvitamin D and Areal Bone Mineral Density in Children with Overweight/Obesity

**DOI:** 10.3390/nu11112760

**Published:** 2019-11-14

**Authors:** Jose J. Gil-Cosano, Luis Gracia-Marco, Esther Ubago-Guisado, Jairo H. Migueles, Jose Mora-Gonzalez, María V. Escolano-Margarit, José Gómez-Vida, José Maldonado, Francisco B. Ortega

**Affiliations:** 1 PROFITH “PROmoting FITness and Health through Physical Activity” Research Group, Sport and Health University Research Institute (iMUDS), Department of Physical Education and Sports, Faculty of Sport Sciences, University of Granada, 18071 Granada, Spain; esther.ubago@gmail.com (E.U.-G.); jairohm@ugr.es (J.H.M.); jmorag@ugr.es (J.M.-G.); ortegaf@ugr.es (F.B.O.); 2 Growth, Exercise, Nutrition and Development Research Group, University of Zaragoza, 50009 Zaragoza, Spain; 3 Universidad de Castilla-La Mancha, Health and Social Research Center, 16002 Cuenca, Spain; 4 Department of Pediatrics, San Cecilio Hospital, 18012 Granada, Spain; mv.escolano@hotmail.com (M.V.E.-M.); gomezvida@gmail.com (J.G.-V.); 5 Department of Pediatrics, School of Medicine, University of Granada, 18016 Granada, Spain; jmaldon@ugr.es; 6 The Institute of Biomedicine Research (Instituto de Investigación Biosanitaria (IBS), 18014 Granada, Spain

**Keywords:** Vitamin D, strength, bone health, mediation, childhood, obesity

## Abstract

The association between vitamin D [25(OH)D] and bone health has been widely studied in children. Given that 25(OH)D and bone health are associated with muscular fitness, this could be the cornerstone to understand this relationship. Hence, the purpose of this work was to examine if the relation between 25(OH)D and areal bone mineral density (aBMD) was mediated by muscular fitness in children with overweight/obesity. Eighty-one children (8-11 years, 53 boys) with overweight/obesity were included. Body composition was measured with dual energy X-ray Absorptiometry (DXA), 25(OH)D was measured in plasma samples and muscular fitness was assessed by handgrip and standing long jump tests (averaged z-scores were used to represent overall muscular fitness). Simple mediation analyses controlling for sex, years from peak height velocity, lean mass and season were carried out. Our results showed that muscular fitness z-score, handgrip strength and standing long jump acted as mediators in the relationship between 25(OH)D and aBMD outcomes (percentages of mediation ranged from 49.6% to 68.3%). In conclusion, muscular fitness mediates the association of 25(OH)D with aBMD in children with overweight/obesity. Therefore, 25(OH)D benefits to bone health could be dependent on muscular fitness in young ages.

## 1. Introduction

The World Health Organization defines osteoporosis as a systemic skeletal disease characterized by low bone density and microarchitectural deterioration of bone tissue [1]. Acquiring an optimal bone mineral accrual during childhood (i.e., late childhood and peripubertal years) is considered an important factor for reducing the risk of osteoporosis later in life [2]. In general, children with overweight/obesity usually have a greater areal bone mineral density (aBMD) than normal-weight children as they mature earlier, tend to be taller and have greater lean mass [2]. Notwithstanding, Rokoff et al. [3] recently showed central adiposity to be inversely associated with aBMD *Z*-score at the total body less head (TBLH) in children with high levels of abdominal fat.

Childhood obesity is associated with a deficient 25(OH)D status in Spain [4]. Vitamin D status is reflected by 25-hydroxyvitamin D (25(OH)D) levels and its concentration in children with obesity is influenced by vitamin D intake, season, ethnicity/race, decreased exposure to sunlight as a consequence of the sedentary lifestyle, or by 25(OH)D sequestration through adipose tissue [5]. This prohormone is essential for bone development and remodeling processes, as well as for normal calcium and phosphorus homeostasis [6]. Some studies evidenced that 25(OH)D-deficient children had lower aBMD *Z*-score at the lumbar spine (LS) and the total body, probably influenced by the consequent increase in parathormone levels [7,8].

Moderate-to-high muscular fitness at a young age is a powerful determinant of health [9]. In this regard, Torres-Costoso et al. [10] found that children with good performance in handgrip and standing long jump had better and worse bone health, respectively. The latter associations were fully mediated by lean mass, whose function seems to be influenced by 25(OH)D levels [11]. When calcitriol (1,25(OH)_2_D, an active metabolite of vitamin D) activates the nuclear vitamin D receptor (VDR), several slow pathways are activated leading to cytoskeletal protein synthesis important for muscle function (i.e., calmodulin, calbindin D-9K or insulin-like growth factor binding protein-3) [12,13,14]. Moreover, the activation of the nuclear VDR also increases phosphate metabolism via increases in the uptake and accumulation of phosphate and ATP, resulting in positive effects on muscle contraction [15]. In addition, the 1,25(OH)_2_D activation of the membranous VDR stimulates rapid actions that affect Ca^2+^ handling and muscle cell proliferation and differentiation [16].

Although the relationship between 25(OH)D and muscular fitness has been described in youth, no study has jointly examined the association of these predictors with aBMD outcomes. Most published studies have been conducted using statistical multivariate procedures in order to control for potential confounders, but these statistical procedures are unable to distinguish between confounding and mediating variables. Mediation analysis allows us to clarify the process underlying the relationship between two variables and the extent to which this relationship can be modified or confounded by a third variable [17]. Therefore, the aim of this study was to examine whether the relationship between 25(OH)D and aBMD outcomes is mediated by muscular fitness in children with overweight/obesity.

## 2. Materials and Methods

### 2.1. Design

A cross-sectional analysis was conducted of the baseline measurements of the ActiveBrains project (registered at Clinicaltrials.gov, number NCT02295072). A detailed description of the study has been published elsewhere [18]. The ActiveBrains project measured 110 children with overweight/obesity aged 8–11 years from Granada (south of Spain) according to the following inclusion criteria: (1) to be overweight or obese based on the World Obesity Federation (formerly named International Obesity Task Force) cut-off points (2) to be 8 to 11 years old, (3) not to have any physical disabilities or neurological disorder that affects their physical performance, and (4) in the case of girls, not to have started the menstruation at the moment of the assessments.

A total of 81 children with overweight/obesity (10.0 ± 1.2 years old, 65% boys) with valid data on 25(OH)D, muscular fitness variables, body composition (i.e., bone, fat and lean mass) and sexual maturation were included in this report. Participants were recruited from the Pediatric Unit of the “San Cecilio” and “Virgen de las Nieves” University Hospitals in the province of Granada, Spain. Furthermore, we contacted several schools of Granada and we advertised the study in the local media, inviting any child meeting the inclusion criteria. The study protocol was approved by the Ethics Committee on Human Research (CEIH) of the University of Granada (Reference: 848, February 2014). Written consent was obtained from parents for the participation of their children.

### 2.2. Measures

#### 2.2.1. Anthropometrics and Sexual Maturation

Participants were weighed using an electronic scale (SECA 861, Hamburg, Germany) with an accuracy of 100 g. A precision stadiometer was used to assess height (cm) and sitting height (SECA 225, Hamburg, Germany) to the nearest 0.1 cm. BMI was calculated as body mass (kg)/height (m^2^) and the participants were classified as overweight or obese according to sex- and age-specific BMI cut-offs defined by Cole et al. [19].

Somatic maturity offset was assessed as years from peak height velocity (PHV) from age, height and sitting height using validated algorithms for children [20]. In boys: −8.128741 + (0.0070346 × (age × sitting height)), where *R*^2^ = 0.906 and the standard error of the estimate = 0.514. In girls: −7.709133 + (0.0042232 × (age × height)), where *R*^2^ = 0.898 and the standard error of the estimate = 0.528. PHV is the period of time of maximum growth in stature and therefore, years from PHV are considered in terms of time before and time after the PHV.

#### 2.2.2. Vitamin D

Venous blood samples were obtained between 8:00 a.m. and 9:00 a.m. by venipuncture after an overnight fast (at least 12 h) from September 2015 to February 2016 (Autumn and Winter). Blood samples in tubes containing EDTA were spun immediately at 30,000 g for 10 min. Plasma was isolated and stored at −80 °C until assayed. Plasma 25(OH)D was analyzed by immunoturbidimetry (Alinity i 25-OH Vitamin D Reagent Kit ref. 08P4522, Abbot, IL, USA) with a sensitivity of 3.5 ng/mL and an intra-assay coefficient of variation of 2.5%.

#### 2.2.3. Muscular Fitness

Upper-body muscular fitness was assessed using the handgrip strength test through a dynamometer with an adjustable grip (TKK 5101 Grip D, Takey, Tokyo Japan). Participants were instructed to squeeze continuously for ≥2 s with the elbow in full extension position. The test was repeated twice (right and left hands alternately). The best score of the 2 attempts for each hand was chosen and averaged [21]. Finally, relative upper-body muscular fitness was expressed per kg of body mass (Handgrip strength (kg/kg)). Lower-body muscular fitness was assessed by the standing long jump test. Participants were instructed to push off vigorously and jump as far forward as possible, trying to land on both feet. The distance reached was taken in centimeters from the take-off line and the heel of the nearest foot at landing. The longest attempt from 3 was recorded (cm). The scientific rationale for the selection of these tests, as well as their validity and reliability, has previously been demonstrated in children and adolescents [21].

A muscular fitness score (muscular fitness *z*-score) was computed by combining the standardized values of handgrip strength (kg/kg) and standing long jump (cm). Each of these variables was standardized as follows: *z*-score = (*i*th value − mean)/SD. The muscular fitness *z*-score was calculated as the mean of the 2 standardized scores (handgrip strength and standing long jump).

#### 2.2.4. Body Composition

Children were scanned with dual-energy X-ray Absorptiometry (DXA) using the Hologic Discovery Wi (Hologic Series Discovery QDR, Bedford, MA, USA). The DXA equipment was calibrated at the start of each testing day by using a lumbar spine phantom as recommended by the manufacturer. All DXA scans and analyses were performed using the GE encore software (version 4.0.2) following the same protocol by the same researcher. The positioning of the participants and the analyses of the results were undertaken following recommendations from the International Society of Clinical Densitometry [22]. The total body scan was used to obtain fat mass, lean mass, and aBMD at the TBLH, arms, and legs.

### 2.3. Statistical Analysis

Descriptive characteristics of the participants are presented as mean ± standard deviation (SD) or percentages. All variables were checked for normality using visual check of histograms, Q-Q and box plots. Interaction analyses were performed for sex and since no significant interactions were found (*p* ≤ 0.28), analyses were performed for boys and girls together.

A partial correlation analysis controlling for sex and years from PHV was performed to examine the relationship between 25(OH)D, muscular fitness variables, TBLH lean mass, and TBLH fat mass.

**Figure 1:**
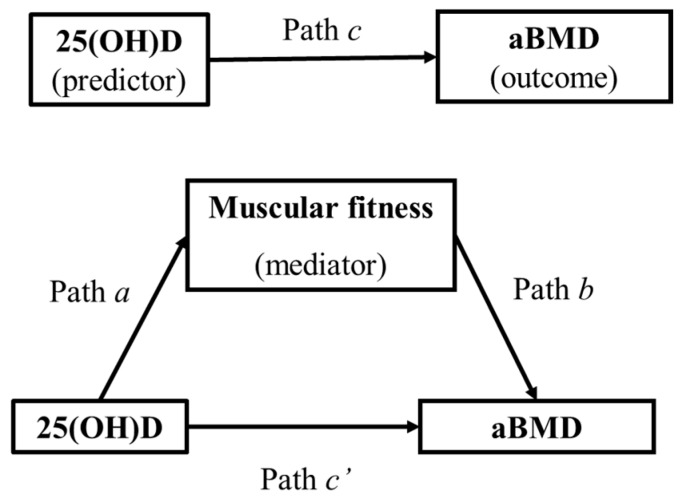
Causal diagram reflecting the simple mediation analyses. Path c shows the association between the predictor and the outcome. Arrows a  ×  b show the natural indirect effect pathway, and c′ shows the natural direct effect pathway. *aBMD:* areal bone mineral density.

We carried out a mediation analysis controlling for sex, years from PHV, TBLH lean mass and season to test whether the association between 25(OH)D and aBMD outcomes was mediated by muscular fitness. These covariates were selected because of their well-known association with aBMD [23,24]. The PROCESS macro version 3.1, model 4, with 10,000 bias-corrected bootstrap samples and 95% confidence intervals was used for these analyses. In a nutshell, the mediation analysis is composed of ordinary least squared regression-based equations (paths) that allow us to answer the question of how a predictor transmits its effect (total effect) on an outcome being partitioned into direct (c’ path) and indirect effect (a × b path). Most contemporary analysts focus on the indirect effect by stating 2 steps in establishing mediation [25]: (1) show that the causal variable is correlated with the mediator (path a); (2) show that the mediator affects the outcome variable controlling for the predictor (path b). Thus, mediation is assessed by the indirect effect of the 25(OH)D (predictor) on aBMD (outcome) through muscular fitness (mediator). The total (c path), direct (c′ path), and indirect effects (a × b paths) are presented (Figure 1). Indirect effects with confidence intervals not including zero were interpreted as statistically significant [25] regardless of the significance of the total effect (the effect of 25(OH)D on aBMD outcomes) and the direct effect (the effect on aBMD outcomes when both 25(OH)D and muscular fitness are included as independent variables). The percentage of mediation (P_M_) was calculated as “(indirect effect/total effect) × 100” to know how much of the total effect was explained by the mediation when the following assumptions were achieved: the total effect is larger than the indirect effect and of the same sign. All the analyses were performed using the IBM SPSS Statistics for Windows version 20.0 (IBM Corp: Armonk, NY, USA), and the level of significance was set to *p* < 0.05.

## 3. Results

Table 1 shows the raw descriptive characteristics of the participants at baseline (mean ± SD). Briefly, the mean age of the participants was 10.0 ± 1.2 years and they were 2.4 ± 0.9 years below PHV, overweight and obesity was evident in 28.4% and 71.6% of them, respectively; the mean 25(OH)D concentration was 31.5 nmol/L and only 6.2% of the children measured fell above the suggested cut-off of 50 nmol/L [26].

Partial correlations between 25(OH)D, muscular fitness variables, TBLH fat mass and TBLH lean mass after adjustment for sex and years from PHV are presented in Table 2. 25(OH)D was positively correlated with muscular fitness *z*-score and handgrip strength (*r* = 0.28 and *r* = 0.29, respectively). Muscular fitness *z*-score was positively correlated with TBLH aBMD and arms aBMD (*r* = 0.24 and r = 0.35, respectively), whilst handgrip strength was positively correlated with arms aBMD (*r* = 0.32). Finally, standing long jump was positively correlated with aBMD at TBLH, arms and legs (*r* = 0.27, *r* = 0.29 and *r* = 0.23, respectively).

### Mediation Analysis

**Figure 2:**
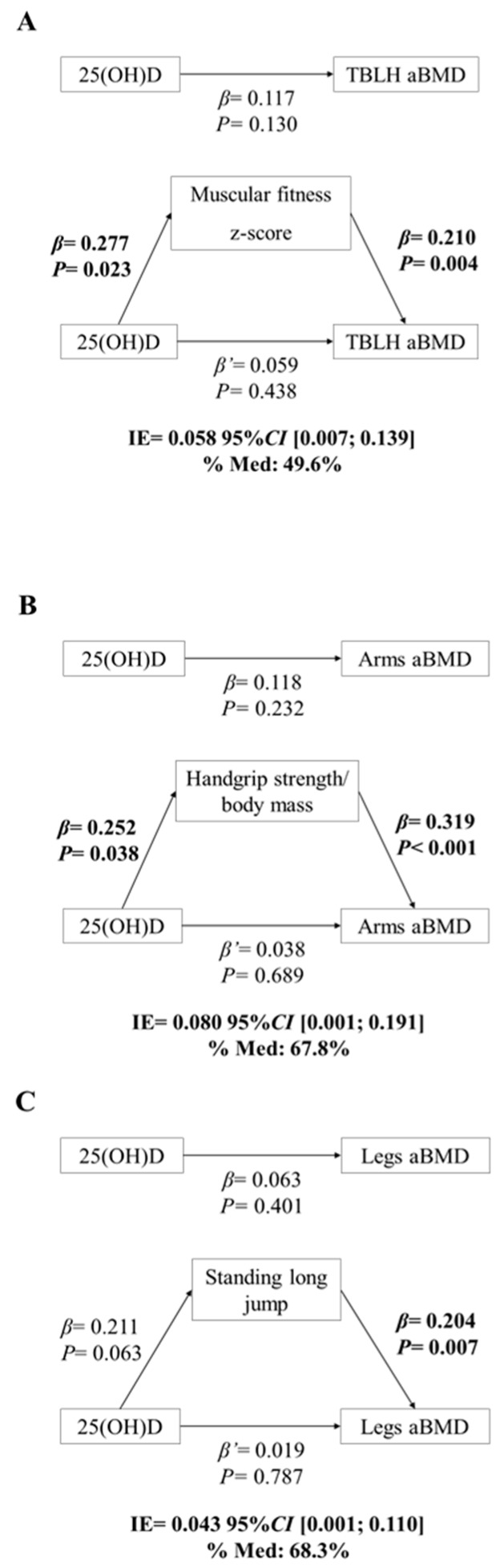
Simple mediation models of the relationship between 25(OH)D and aBMD outcomes using muscular fitness as a mediator, controlling for sex, years from PHV, TBLH lean mass and season. Muscular fitness *z*-score was used as a mediator in panel (**A**), handgrip strength/body mass was used as mediator in panel (**B**) and standing long jump was used as a mediator in panel (**C**). *Z*-score mean computed from handgrip strength (kg/kg) and standing long jump (cm) tests; *PHV* peak height velocity; *TBLH* total body less head; *25(OH)D* 25-hydroxyvitamin D; *aBMD* areal bone mineral density

Mediation analysis models are depicted in Figure 2. 25(OH)D was not significantly associated with any of the aBMD outcomes (c, total effect). Regarding path a, 25(OH)D was positively associated with muscular fitness *z*-score (Figure 2A, β = 0.277, *p* = 0.023) and handgrip strength (Figure 2B, β = 0.252, *p* = 0.038). In the path b, in all mediation models, muscular fitness was positively associated with TBLH aBMD (Figure 2A, β = 0.210, *p* = 0.004), arms aBMD (Figure 2B, β = 0.319, *p* < 0.001) and legs aBMD (Figure 2C, β = 0.204, *p* = 0.007). Finally, when 25(OH)D and muscular fitness were simultaneously included as independent variables (c’, direct effect), aBMD outcomes were not predicted. There was a significant mediating effect of muscular fitness on the relationship of 25(OH)D with TBLH aBMD, arms aBMD and legs aBMD (P_M_ ranged from 49.6 to 68.3%).

## 4. Discussion

In the present study, we revealed a mediating effect of muscular fitness on the relationship between 25(OH)D levels and aBMD at the TBLH, arms, and legs after controlling for sex, years from PHV, TBLH lean mass and season. To the best of our knowledge, this is the first study in children with overweight/obesity analyzing whether muscular fitness acts as mediator in the association between 25(OH)D and aBMD outcomes.

Our results show no significant association between 25(OH)D and aBMD outcomes after adjusting for sex, years from PHV, TBLH lean mass and season (path c, total effect). This finding agrees with Hauksson et al. [27] who found no significant association between 25(OH)D levels and bone mineral accrual in Icelandic children at ages 7 and 9. On the contrary, Pekkinen et al. [7] reported that 25(OH)D status was a key determinant of aBMD in children and adolescents. In this regard, 25(OH)D status has been highlighted as a significant predictor of peak bone mass in males but not in females during childhood [28]. Likewise, non-significant associations between 25(OH)D and bone outcomes have been reported in American prepubertal girls after adjusting for potential cofounders [29] and in Finnish prepubertal girls after adjustment for maturation and BMI [8]. This could be explained by the differences in sex hormone effects on bone since estrogens may counteract the effects of lower 25(OH)D levels in females, whereas in males this compensatory effect is absent [28]. Nevertheless, we did not find sex interaction between 25(OH)D and aBMD outcomes, suggesting that these sex differences in hormonal effects on bone might not occur in prepubertal children with overweight/obesity since estradiol levels may be high in both boys and girls [30,31].

A few studies have assessed the effect of 25(OH)D in relation to muscular fitness in children [32,33,34]. In addition, the present study does so, taking into account different ways of measuring muscular fitness in the upper and lower limbs. The results of the present investigation confirm a relationship between 25(OH)D levels and muscular fitness *z*-score, handgrip strength and standing long jump (path a). These results agree with Foo et al. [32] who observed that adolescent girls with sufficient 25(OH)D status performed significantly better in handgrip strength compared with those with deficient or severely deficient status. Moreover, our results partly concur with Ward et al. [33] who found a positive association between 25(OH)D levels and the performance in countermovement jump in British adolescent girls. Otherwise, a study carried out with children did not find any relationship between handgrip strength and 25(OH)D status [34].

In this study, muscular fitness *z*-score, handgrip strength, and standing long jump were positively associated with TBLH aBMD, arms aBMD and legs aBMD, respectively (path b). Torres-Costoso et al. [10] reported a positive association between handgrip strength and aBMD outcomes in children aged 8–11 years, although a negative association between standing long jump and aBMD outcomes was found. The latter inverse association contrasts with our results. A possible explanation for these differences could be the different weight status of the participants included in both studies (BMI, 18.8 ± 3.8 vs. 26.3 ± 3.4). In addition, the fact that our results were adjusted for TBLH lean mass (but not in Torres-Costoso’s study) could modify the direction of the association. Our findings agree with the literature and support the fact that bones adapt their resistance to the mechanical stimuli (i.e., body mass and muscle contractions) placed on them [35]. Moreover, it should be noted that the performance in the standing long jump test may be affected by the coordination skills [9], which might not be fully developed in 8–11-year-old children.

Our results show that the total effect of 25(OH)D on aBMD outcomes was mediated by muscular fitness *z*-score, handgrip strength and standing long jump (P_M_ ranged from 49.6 to 68.3%). Since the mediation analysis assumes that the predictor variable causes the mediator [17], muscular fitness may be an intermediate step in the causal pathway of 25(OH)D with aBMD. There is consistent evidence regarding the bivariate association of muscular fitness with both 25(OH)D [36] and aBMD outcomes [37,38]. Otherwise, the relationship between 25(OH)D and bone in children remains controversial [27,32]. In addition, a recent study has reported that the association between muscular fitness and aBMD is fully mediated by lean mass, whose function appears to be affected by 25(OH)D levels [11]. Together with our results, this evidence indicates that increasing 25(OH)D levels may increase muscular fitness and, ultimately, the aBMD. As an optimal bone mineral accrual is critical during childhood in order to prevent osteoporosis later in life [2], public health policies should start at an early age. Therefore, school-based interventions aiming at improving outdoor physical activity levels are justified among children to synthesize 25(OH)D and, ultimately, improve muscular fitness.

### Strengths and Limitations

The current study has several limitations that should be acknowledged. First, our cross-sectional design rules out the possibility of identifying cause-effect relationships. Thus, the reported findings need to be confirmed prospectively. Second, the number of participants with complete data in all studied variables is relatively small. Third, although we did not find interaction by sex, our results need to be confirmed by studying boys and girls separately. Finally, calcium intake was not available and therefore, we did not include it in the model as a cofounder (i.e., vitamin D interacts with calcium affecting bone health [6]).

The present study has also several strengths, such as the use of relevant sets of cofounders (i.e., sex, years from PHV, TBLH lean mass and season) that are crucial to analyze the association of 25(OH)D with bone outcomes in children. Furthermore, valid and reliable tests for assessing muscular fitness were chosen from the ALPHA-Fitness battery [21]. Finally, we used DXA for assessing aBMD bone outcomes which are the gold standard for measuring bone outcomes and have been used worldwide in the pediatric population [22].

## 5. Conclusions

Muscular fitness plays a key role in the relationship between 25(OH)D levels and aBMD at the TBLH and arms. Increasing 25(OH)D levels may improve muscular fitness and, ultimately, aBMD in children with overweight/obesity. Future longitudinal studies must be conducted in order to confirm these findings.

## Figures and Tables

**Table 1:** Characteristics of the study sample by sex.

Variables	All (*n* = 81)	Boys (*n* = 53)	Girls (*n* = 28)
Age (years)	10.0 ± 1.2	10.2 ± 1.2	9.7 ± 1.2
Years from PHV (years)	−2.4 ± 0.9	−2.6 ± 0.9	−1.8 ± 1.1
Height (cm)	143.9 ± 8.7	144.5 ± 8.1	142.7 ± 9.8
Body mass (kg)	54.8 ± 10.7	55.8 ± 10.7	53.1 ± 10.8
TBLH fat mass (kg) ^a^	21.9 ± 5.8	22.1 ± 5.9	21.5 ± 5.8
TBLH lean mass (kg) ^a^	26.6 ± 5.2	27.3 ± 4.9	25.5 ± 5.3
BMI (kg·m^−2^)	26.3 ± 3.4	26.5 ± 3.4	25.9 ± 3.3
Overweight (%)	28.4	26.4	32.1
Obesity (%)	71.6	73.6	67.9
Autumn (%)	91.4	90.6	92.9
Winter (%)	8.6	9.4	7.1
25(OH)D (nmol/L) ^a,^*	31.5 ± 9.5	32.7 ± 9.6	29.2 ± 8.9
Deficiency (%)	46.9	43.4	53.6
Insufficiency (%)	46.9	49.1	42.9
Sufficiency (%)	6.2	7.5	3.6
Muscular fitness *z*-score ^b^	0.000 ± 1.000	0.032 ± 0.098	−0.061 ± 1.037
Handgrip strength (kg)/body mass (kg) ^a^	0.307 ± 0.059	0.309 ± 0.058	0.303 ± 0.059
Standing long jump (cm) ^a^	106.2 ± 17.8	106.5 ± 17.9	105.7 ± 17.9
TBLH (g·m^−2^) ^a^	0.772 ± 0.059	0.775 ± 0.059	0.766 ± 0.058
Arms (g·m^−2^) ^a^	0.607 ± 0.041	0.613 ± 0.041	0.596 ± 0.040
Legs (g·m^−2^) ^a^	0.913 ± 0.079	0.917 ± 0.082	0.906 ± 0.074

PHV peak height velocity; TBLH total body less head; BMI body mass index; 25(OH)D 25-hydroxyvitamin D; aBMD areal bone mineral density; ^a^ Values were Blom-transformed before analysis, but non-transformed values are presented; ^b^ *Z*-score mean computed from handgrip strength (kg/kg) and standing long jump (cm) tests; * Vitamin D status was defined as follows [26]: Sufficiency, > 50 nmol·L^−1^; Insufficiency, 30–50 nmol/L^−1^; Deficiency, <30 nmol/L^−1^.

**Table 2:** Partial coefficients of the independent variable with muscular fitness variables and aBMD outcomes adjusted for sex and years from PHV.

	Muscular Fitness *z*-Score ^b^	Handgrip Strength/Body Mass	Standing Long Jump	TBLH aBMD	Arms aBMD	Legs aBMD
25(OH)D	0.275 *	0.285 *	0.186	0.039	0.043	−0.011
Muscular fitness z-score ^b^	-	0.881 **	0.869 **	0.244 *	0.352 *	0.182
Handgrip strength/body mass		-	0.540 **	0.165	0.320 *	0.089
Standing long jump			-	0.266 *	0.295 *	0.233 *
TBLH aBMD				-	0.764 **	0.894 **
Arms aBMD					-	0.577 **

*PHV* peak height velocity; 25(*OH*)*D* 25-hydroxyvitamin D; *TBLH* total body less head: *aBMD* areal bone mineral density; ^b^ *Z*-score mean computed from handgrip strength (kg/kg) and standing long jump (cm) tests; Boldface indicates statistical significance: * *p* < 0.050, ** *p* < 0.001.
